# Stepwise targeting and responsive lipid-coated nanoparticles for enhanced tumor cell sensitivity and hepatocellular carcinoma therapy

**DOI:** 10.7150/thno.42008

**Published:** 2020-02-19

**Authors:** Ying Li, Yunqiu Miao, Mingshu Chen, Xi Chen, Feifei Li, Xinxin Zhang, Yong Gan

**Affiliations:** 1 Department of Pharmacy, School of Medicine, Shenzhen University, Shenzhen 518060, China; 2 Shanghai Institute of Materia Medica, Chinese Academy of Sciences, 501 Haike Road, Shanghai 201203, China; 3 School of Pharmacy, Shanghai University of Traditional Chinese Medicine, Shanghai 201203, China

**Keywords:** drug delivery, pH sensitive, charge conversion, tumor targeting, hepatocellular carcinoma

## Abstract

**Rationale:** Antitumor drug delivery faces multiple barriers that require consecutively achieving tumor targeting, selective cellular uptake and sufficient intracellular drug dosage.

**Methods:** Herein, we designed smart nanoparticles (GPDC-MSNs) that can accumulate stepwise in tumor tissues, selectively enter cancer cells by responding to the acidic tumor extracellular environment, and achieve considerable drug release in the intracellular microenvironment. The GPDC-MSNs comprise the synthesized material galactosyl-conjugated PEO-PPO-PEO (Gal-P123) for hepatocellular carcinoma (HCC) targeting, the tumor extracellular pH-responsive lipid (2E)-4-(dioleostearin)-amino-4-carbonyl-2-butenonic (DC) for selective cellular internalization, and antitumor drug irinotecan (CPT-11)-loaded mesoporous silica nanoparticles (MSNs) for on-demand intracellular drug release.

**Results:** GPDC-MSNs are negatively charged at pH 7.4 and promote active HCC targeting mediated by the asialoglycoprotein receptor. Upon reaching the weakly acidic tumor microenvironment, the nanoparticles undergo charge conversion to neutral, enhancing cellular internalization. Moreover, the encapsulated CPT-11 can be retained within GPDC-MSNs in the blood circulation but undergo intracellular burst release, which facilitates the apoptosis of tumor cells. GPDC-MSNs significantly increased HCC selectivity* in vivo* and exhibited up to 25 times higher accumulation in tumor tissue than in normal hepatic tissue, thus achieving superior antitumor efficacy and low systemic toxicity.

**Conclusion:** This stepwise-responsive nanoparticle should serve as a valuable platform and promising strategy for HCC treatment.

## Introduction

Chemotherapy plays a significant role and has been established as a standard treatment for various cancers in recent decades 1, 2. However, free antitumor drugs decentralize to the entire body with low accumulation in tumors, which causes disappointingly low therapeutic efficacy and fatal side effects. Although a variety of nano-based systems have marginally improved drug pharmacokinetics, clinical data reveal that nanomedicines do not exhibit significantly prominent antitumor efficacy in long-term treatments 3. Typically, delivering a nanomedicine to the cytosol of cancer cells in tumor tissues requires overcoming a series of biological barriers to achieve “CAPIR”: includes circulation in the blood, accumulation in the tumor site, deep penetration into avascular tumor tissue, cellular internalization and intracellular drug release 4. Highly efficient tumor tissue accumulation, selective cancer cell internalization, and sufficient intracellular drug release remain the key goals.

A rationally designed nanosystem should address these issues successively. The first requirement of an antitumor drug delivery system is the ability to target tumor sites. Although nanoparticles can accumulate in tumor tissues after intravenous administration due to the enhanced permeation and retention (EPR) effect, this passive targeting strategy may not be effective enough. For example, Doxil (a PEGylated liposomal doxorubicin) has a hydrophilic PEG corona on its surface, which increases intratumoral accumulation compared with that of free doxorubicin, but the target efficiency and the consequent therapeutic efficacy are still far from satisfactory. Therefore, many antibody, peptide, folate and biotin receptors overexpressed in cancer cells are adopted to enhance the efficiency of targeting to tumor sites 5. Galactosyl-conjugated materials, for instance, are widely believed to be promising for hepatocellular carcinoma (HCC) targeting 6. However, existing off-target effects suggest that the modification of target ligands alone is far from sufficient, and selective cellular uptake and effective intracellular dosage must be pursued 7, 8. The selective uptake of nanoparticles into cancer cells can be achieved by utilizing the differential microenvironment between tumors and normal tissues. The unique tumor microenvironment, including acidic pH 9-11, redox potential 12 and unique enzymatic activity 13, offers great opportunities to develop smart stimuli-responsive drug vehicles. Notably, pH responsivity is one of the most extensively exploited since the tumor extracellular environment is more acidic (pH 6.2-6.9) than blood and normal tissues (pH 7.3-7.4) 14. It has been widely acknowledged that cationic materials are more easily internalized and often induce cytotoxicity and opsonization 15; therefore, acid-triggered charge conversion in acidic microenvironments is an effective strategy. Nanoparticles can remain slightly anionic during circulation 16, 17 and be cationized before internalization 18. Our previous studies have reported a pH-sensitive lipid, (2E)-4-(dioleostearin)-amino-4-carbonyl-2-butenonic (DC), that can change its charge from negative to neutral in the acidic tumor environment and enhance cancer cell internalization while mitigating cytotoxicity 19. Moreover, stimuli-triggered rapid drug release at the target site is a promising approach to enhancing therapeutic efficacy in cancer cells. However, this contrasts with a passive delivery system in which cargo release is achieved by simple diffusion and slow degradation of the nanoparticles, which requires a long exposure time to release a satisfactory amount of the drug. Most reported singly responsive nanoparticles have difficulty achieving both tumor site targeting and stimulated intracellular drug release in complex biological systems, often leading to insufficient drug release in tumor sites and thus to reduced therapeutic efficacy 20. Therefore, the development of appropriate hierarchically responsive nanoplatforms is of great value for improving the therapeutic index of drugs 21-23.

**Scheme 1:**
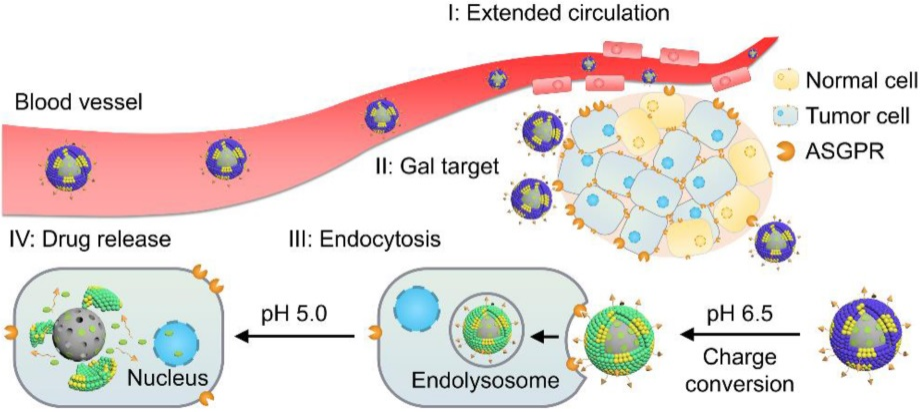
Illustration of the stepwise targeting and responsiveness of the lipid-coated nanoparticles GPDC-MSNs.

Herein, to realize programmed drug delivery, including effective HCC targeting, cellular uptake and sufficient drug release in the cytosol, we designed smart lipid-coated nanoparticles (GPDC-MSNs) in accordance with the transport process *in vivo* (Scheme 1) to combine the abovementioned desired effects. The GPDC-MSNs consist of Gal-P123 (galactosyl-conjugated PEO-PPO-PEO) for HCC targeting, DC for pH-responsive cellular internalization, and antitumor drug irinotecan (CPT-11)-loaded mesoporous silica nanoparticles (MSNs) for on-demand intracellular drug release 24. CPT-11, a DNA topoisomerase-I inhibitor, has been widely used in some solid malignant tumors and studied in clinical trials for its application in HCC 25-27. GPDC-MSNs are expected to undergo multiple-step delivery: (i) the anionic charge supports extended circulation, while the surface galactosyls target HCC; (ii) mediated by DC, the surface potential turns from negative to neutral in the tumor microenvironment (pH 6.5), facilitating cellular uptake; (iii) After internalization, in acidic endolysosomes (pH 5.0), the supported lipid layer dissociates and induces burst release of the antitumor drug CPT-11 from the MSNs. The stepwise targeting and responsive cellular uptake, intracellular drug release, and antitumor efficacy were investigated *in vitro* and *in vivo*.

## Materials and Methods

### Materials

Di-tert-butyl dicarbonate, 3-amino-1,2-propanediol, oleoyl chloride, citraconic anhydride, tetraethyl orthosilicate (TEOS, 98%) and cetyltrimethylammonium bromide (CTAB, >99%) were purchased from Aladdin-chemistry, Co., Ltd (Shanghai, China). Irinotecan hydrochloride (CPT-11, 99.8%) was obtained from Knowshine Pharmachemicals Inc. (Shanghai, China). Phospholipid PC-98T was purchased from A.V.T Co., Ltd (Shanghai, China). Galactosyl conjugated PEO-PPO-PEO (Gal-P123) was synthesized in the previous studies 28. Lipid material (2E)-4-(dioleostearin)-amino-4-carbonyl-2-butenonic (DC) and dioleostearin-3-amino-1, 2-propanediol (DOA) were synthesized as described in our previous report 19. Bafilomycin A1, propidium iodide (PI) and 1,1'-dioctadecyl-3,3,3',3'-tetramethylindotricarbocyanine iodide (DiR) were from Sigma Aldrich (MO, USA). All other reagents were of analytical grade and used as received.

### Preparation of nanoparticles

Mesoporous silica nanoparticles (MSNs) were synthesized by the CTAB-templated, base-catalyzed condensation reaction of TEOS. Briefly, 0.12 g of NaOH, 0.6 g of CTAB and 3.5 mL of trimethylbenzene were dissolved in 400 mL deionized water under vigorous stirring at 80 °C. TEOS (2.5 mL) was added dropwise within 20 min as a silicon source. The reaction mixture was stirred for 24 h and centrifuged to collect the synthesized materials. Then, the organic template was completely removed through refluxing in a mixed solution of ethanol (90 mL) and hydrochloric acid (5 mL, 35%) at 80 °C for 12 h. The resulting MSNs were dried for 12 h at room temperature in vacuum. For drug loading, MSNs were dispersed in an aqueous solution of CPT-11 at a weight ratio of 1:1, followed by incubation at room temperature for 24 h. The free CPT-11 was removed by centrifugation, and the CPT-11-loaded MSNs (CPT-11@MSNs) were collected.

CPT-11-loaded GPDC-MSNs (CPT-11@GPDC-MSNs) were prepared by hydrating a dried lipid film containing 25 mg of DC, 5 mg of Gal-P123 and 20 mg of phospholipid PC-98T with CPT-11@MSN colloidal solution at a ratio of 2:1 (w/w). This was followed by extrusion 8 times through a 100 nm-pore polycarbonate membrane with an Avestin EmulsiFlex-C5 high pressure extruder (Ottawa, Canada). To prepare comparison formulations, CPT-11@GP-MSNs and CPT-11@PDC-MSNs were obtained by the same procedures described above, except that Gal-P123 or PEO-PPO-PEO and DC were used to coat the CPT-11@MSN cores.

For fluorescent labeling, propidium iodide (PI) or DiR fluorescent dye was conjugated with the nanoparticles. In detail, MSNs were dispersed in an aqueous solution containing PI at a weight ratio of 1000:1 and stirred for 24 h at room temperature to label the mesoporous cores of GPDC-MSNs, PDC-MSNs and GP-MSNs. DiR was added to the lipid solution before film formation to label the lipid shells of nanoparticles. The final concentration of DiR in each formulation was 5 μg/ml. The excess fluorescent dye was removed by a Sephadex G15 column.

### Characterization of nanoparticles

The mesostructure ordering of MSNs was characterized by small-angle X-ray diffraction (SAXRD) on a Shimadzu XRD-6000 diffractometer (Kyoto, Japan). The porosities of MSNs were measured via nitrogen adsorption-desorption isotherms at 77 K on a Micromeritics Tristar 3000 analyzer. The morphology and structure of MSNs and GPDC-MSNs were observed via CM-200FEG transmission electron microscopy (TEM) with an accelerating voltage of 200 kV. DC was added to the lipid membrane of GPDC-MSNs at contents of 20, 30, 40, 50, and 60% (w/w), and the particle size and zeta potential (ζ-pot.) were measured using a Malvern Zetasizer Nano ZS analyzer (Worcestershire, UK) to choose the optimal DC ratio in the formulation. The stability of GPDC-MSNs and MSNs under physiological conditions was evaluated by suspending nanoparticles in pH 7.4 PBS containing 10% fetal bovine serum. The samples were kept at 25 °C, and the sizes were measured by dynamic light scattering (DLS) at predetermined time intervals. To evaluate the drug loading efficiency, CPT-11-loaded GPDC-MSNs and MSNs were suspended in methanol and sonicated thoroughly to remove the CPT-11 from the nanoparticles. After centrifugation, the supernatant was collected, and the concentration of CPT-11 in the supernatant was determined using a Biotec Synergy H1 microplate reader at an excitation wavelength of 368 nm and an emission wavelength of 420 nm. The drug loading efficiency (DL%) of the nanoparticles was calculated as the mass of the loaded drug divided by the protein mass of the CPT-11-loaded nanoparticles. In addition, the Gal-P123 content in the lipid membrane of GPDC-MSNs was studied by the cellular uptake in Huh-7 cells, and the internalized nanoparticles were detected by flow cytometry. The details of the cellular uptake method are described in the following section.

### Acidic pH-triggered charge conversion

GPDC-MSNs or GP-MSNs (without lipid DCs) were incubated in PBS buffers of different pH (7.0, 6.5, 6.0, 5.5 and 5.0) for 2 h at 37 °C. Samples were removed to measure zeta potential and diluted with methanol and fluorescamine acetone solution to determine the exposed amines. After incubation in the dark at room temperature for 10 min, the fluorescence intensity was assayed at an excitation wavelength of 382 nm and an emission wavelength of 474 nm using a Biotec Synergy H1 microplate reader. One hundred percent exposed amine was calculated from the fluorescence of DOA (the synthetic intermediate of DC), and 0% was calculated from the fluorescence of blank buffer solution as a negative control. Amino convention efficiency% = (A - A_PBS_) / A_DOA_ × 100%.

### Cell and multicellular tumor spheroid (MCS) cultures

The human hepatoma cell line Huh-7 with high expression of asialoglycoprotein (ASGP) receptor and the human normal liver cell line LO2 were cultured in DMEM containing 10% fetal bovine serum (Gibco, USA), 100 U/mL penicillin and streptomycin in a humidified 5% CO_2_ incubator at 37 °C.

The Huh-7 MCSs were cultured as described previously 29. Briefly, Huh-7 cells were digested, centrifuged and resuspended in fresh cell culture medium to obtain a single-cell suspension. Then, the Huh-7 single-cell suspension (1,000 cells) was transferred into a flat-bottomed 48-well plate precoated with 1% agarose (w/v) and cultured in the medium for 1 week.

### Cellular uptake and penetration efficiency of nanoparticles

To evaluate the cellular uptake of nanoparticles, PI-labeled GPDC-MSNs, PDC-MSNs and GP-MSNs were prepared. For imaging, Huh-7 and LO2 cells were seeded on coverslips in 24-well plates at a density of 10^4^ cells/well and cultured for 24 h. The cells were then incubated with PI-labeled nanoparticles, and fluorescamine was added to label the exposed amino groups of DCs after hydrolysis. The pH of the culture medium was adjusted to 7.4 or 6.5 to simulate the physiological or tumor microenvironment. After 4 h of incubation, the culture medium was removed, and the cells were washed and fixed. The intracellular distribution of nanoparticles was observed using an Olympus FluoView FV1000 confocal laser scanning microscope (CLSM). For flow cytometric analysis, Huh-7 and LO2 cells were incubated with PI-labeled nanoparticles for 4 h. Thereafter, the cells were washed and detached for data collection using a BD FACS flow cytometer. To observe the inhibitory effect of galactose on the receptor-mediated endocytosis of nanoparticles, Huh-7 cells were incubated with 20 mM galactose in pH 7.4 medium for 1 h prior to treatment with PI-labeled nanoparticles. The internalized nanoparticles were detected by CLSM and flow cytometry as previously described.

MCSs were used to study the tumor penetration ability of nanoparticles. PI-labeled GPDC-MSNs, PDC-MSNs and GP-MSNs were cocultured with MCSs in culture medium (pH 6.5) for 4 h, gently washed with PBS and then observed by CLSM. The fluorescence intensity of nanoparticles in MCSs was analyzed with ImageJ software.

### Drug release triggered by pH

Drug release from nanoparticles was investigated in PBS at different pH values (pH 7.4 and 5.0). Briefly, CPT-11-loaded MSNs, GP-MSNs, PDC-MSNs and GPDC-MSNs were sealed into dialysis tubes (MWCO 14,000 Da), submerged in PBS and shaken at a speed of 100 rpm at 37 °C. At desired time intervals, samples were withdrawn from the outer medium, and the solution was immediately replenished with an equal volume of fresh PBS. The amount of drug released was determined by HPLC. To further study the pH-triggered release procedure of CPT-11@GPDC-MSNs, nanoparticles were added to dialysis bags, which were immersed in simulated body fluid (PBS, pH 7.4), tumor microenvironment conditions (PBS, pH 6.5) and endolysosomal acidic conditions (PBS, pH 5.0). CPT-11 release from GPDC-MSNs was measured as described above.

For real-time observation of intracellular cargo release from GPDC-MSNs, Huh-7 cells were seeded into a standard glass-bottom Petri dish (22 mm in diameter, GWSt-3522 WillCo-dish) at a density of 10^4^ cells/well. PI-labeled GPDC-MSNs and fluorescamine were added to the dish, and then the pH of the culture medium was adjusted from pH 7.4 to 7.2, 7.0, 6.8 and 6.5 at 20 min intervals. Images were acquired using CLSM. The effect of endosomal acidification on the intracellular release of PI-labeled GPDC-MSNs was studied by adding an additional 200 nM bafilomycin A1 to the culture medium (pH 6.5).

To study the intracellular release of CPT-11 formulations, Huh-7 cells with a density of 10^5^ cells/well were seeded in 24-well plates for 24 h. The cells were then incubated with free CPT-11, CPT-11 loaded GP-MSNs, PDC-MSNs or GPDC-MSNs in culture medium (pH 6.5). At predetermined intervals, the cells were washed, detached and lysed. The intracellular amount of CPT-11 was measured using a Biotec Synergy H1 microplate reader at an excitation wavelength of 368 nm and an emission wavelength of 420 nm.

### Cytotoxicity and cell apoptosis

For the* in vitro* cytotoxicity study, Huh-7 cells were seeded in a 96-well plate at a density of 10^4^ cells/well and cultured overnight. Free CPT-11- and CPT-11-loaded GP-MSNs, PDC-MSNs and GPDC-MSNs at various drug concentrations were incubated with the cells for 72 h in culture medium (pH 7.4 or 6.5), after which the MTT assay was performed and measured at 490 nm by a microplate reader.

For quantitative measurement of apoptosis, cells were treated with formulations containing 40 μg/mL CPT-11 for 24 h, harvested and washed with PBS. Then, the Annexin V-FITC Apoptosis Detection Kit was applied to quantify the apoptotic cells by a standard FACS assay.

### Pharmacokinetic study

Male Sprague-Dawley (SD) rats (200 ± 20 g) were obtained from Shanghai Laboratory Animal Research Center. All animal experiments were performed in accordance with NIH guidelines and approved by the Institutional Animal Care and Use Committee at Shanghai Institute of Materia Medica. Free CPT-11, CPT-11-loaded GPDC-MSNs, PDC-MSNs and GP-MSNs were intravenously (i.v.) injected into SD rats via the tail vein at a CPT-11 dose of 10 mg/kg, followed by the collection of blood samples at predetermined time intervals. After separation of the plasma fraction, the drug was extracted with an acidic acetonitrile solution (0.1 mol/L phosphoric acid/acetonitrile, 1:5 v/v). The CPT-11 concentration was measured by HPLC using a mobile phase consisting of a 23/77 (v/v) mixture of acetonitrile and 5 mM potassium dihydrogen phosphate solution at a flow rate of 1.0 mL/min.

### Biodistribution and tumor targeting

BALB/c nude mice were obtained from Shanghai Laboratory Animal Research Center. All animal experiments were performed in accordance with NIH guidelines and approved by the Institutional Animal Care and Use Committee at Shanghai Institute of Materia Medica. Mice were anesthetized by intraperitoneal injection of pentobarbital sodium at a dose of 45 mg/kg, and the abdomen was opened via a midline incision to expose the liver. Then, 10^7^ Huh-7 cells in 100 μL were implanted into a lobe of the liver to establish an orthotopic tumor xenograft model. Fourteen days after Huh-7 cell implantation, the mice were intravenously injected with free DiR, DiR-labeled GPDC-MSNs, PDC-MSNs and GP-MSNs. At predetermined intervals post injection, the mice were anesthetized and visualized in an IVIS Spectrum CT *in vivo* imaging system (PerkinElmer, USA). The excitation and emission wavelengths were 710 and 760 nm, respectively. Finally, the mice were sacrificed, and the major organs (hearts, livers, spleens, lungs and kidneys) were excised and imaged. The fluorescence intensity in the organs was analyzed with ImageJ software. The orthotopic xenografted tumors in the livers were further visualized using micro CT imaging in the IVIS Spectrum CT.

To study the nanoparticle targeting effect, Huh-7 orthotopic tumor xenograft mice were treated with free PI, PI-labeled GPDC-MSNs, PDC-MSNs and GP-MSNs. At 4 h post injection, the mice were sacrificed, and the livers were then excised and frozen in OCT embedding medium. Sections were prepared with a Leica cryotome cryostat and stained with H&E. The slices were observed using an Olympus FluoView FV1000 confocal microscope. The fluorescence intensity in the sections was analyzed with ImageJ software.

### *In vivo* antitumor efficacy

The antitumor efficacy of CPT-11-loaded nanoparticles was evaluated in Huh-7 ectopic and orthotopic tumor xenograft mice. Huh-7 ectopic tumor xenograft mice were established by the subcutaneous injection of Huh-7 cells (10^7^) into the right flank of male BALB/c nude mice. When the tumor volume reached approximately 50 mm^3^, the mice were randomly divided into six groups (n=6) and intravenously injected with saline, a low dose (10 mg/kg) or a high dose (100 mg/kg) of free CPT-11, CPT-11-loaded GPDC-MSNs, PDC-MSNs or GP-MSNs with a CPT-11 dose of 10 mg/kg every three days. The tumor volume (volume = (length × width^2^)/2) and body weight were recorded over time. To evaluate the antitumor efficacy in Huh-7 orthotopic tumor xenograft mice, Huh-7 cells (10^7^) were implanted into a lobe of the liver and allowed to grow for fourteen days to establish an orthotopic tumor xenograft model (n = 6). The same treatment schedule as above was followed, and survival was defined as natural death. The tumor-bearing mice were sacrificed on day 22, and the livers were excised. Finally, the livers were processed routinely into paraffin, sectioned at a thickness of 8 µm and stained with the TUNEL method with an *In Situ* Cell Death Detection Kit for apoptosis analysis.

## Results and Discussion

### Preparation and characterization of nanoparticles

**Figure 1:**
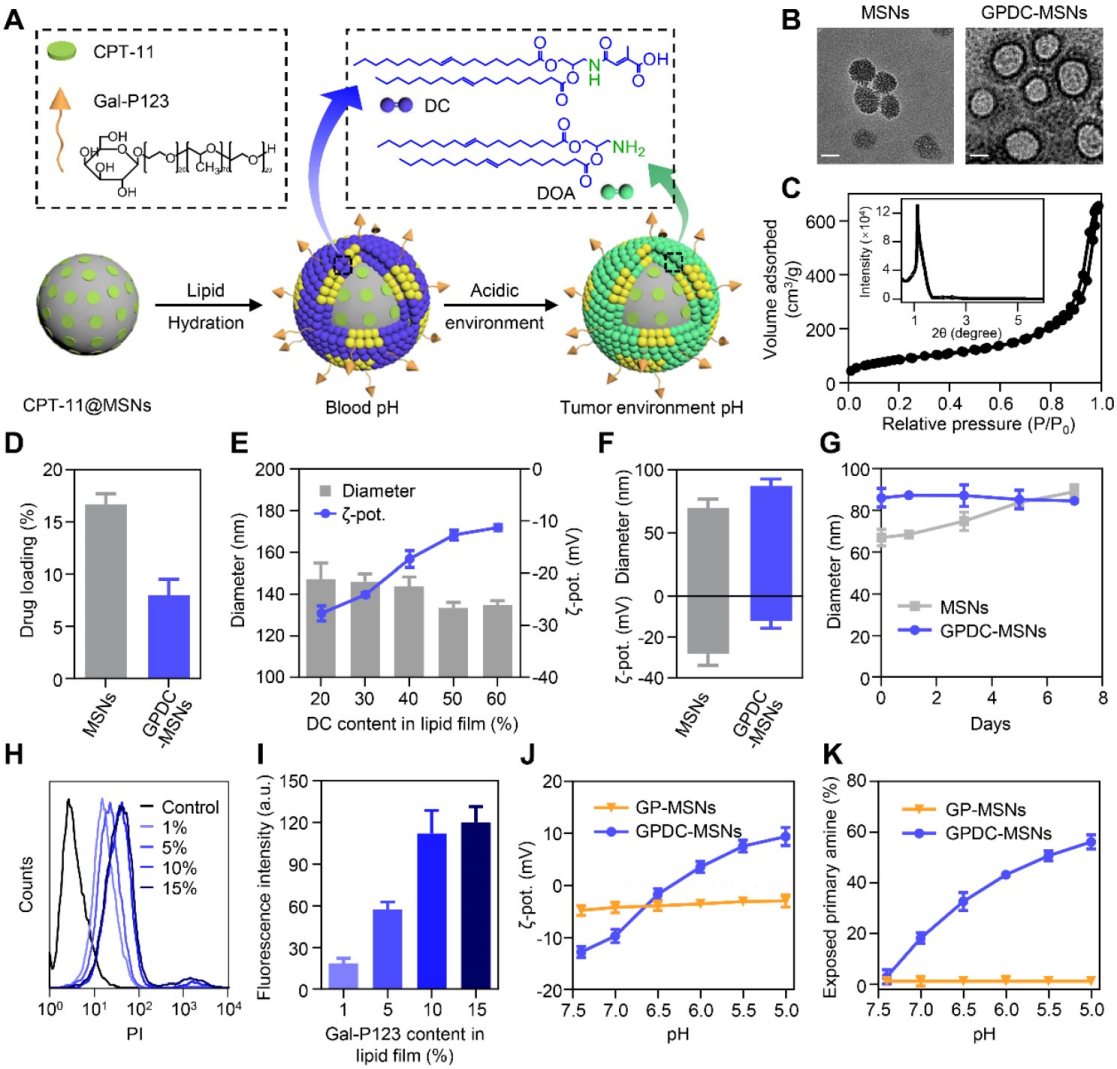
(A) Schematic diagram of the structure and pH sensitivity of CPT-11@GPDC-MSNs. (B) TEM images of MSNs and GPDC-MSNs. Scale bar: 50 nm. (C) SAXRD pattern and nitrogen adsorption-desorption isotherms of the synthetic MSNs. (D) Drug loading efficiency of MSNs and GPDC-MSNs. (E) Particle size and zeta potential of GPDC-MSNs with various DC content in lipid film. (F) Particle size, zeta potential and (G) stability of MSNs and GPDC-MSNs. (H) Representative flow cytometry histograms and (I) quantitative fluorescence intensities of Huh-7 cells after incubation with GPDC-MSNs with various Gal-P123 content. (J) Zeta potential changes and (K) exposed primary amines of GPDC-MSNs and GP-MSNs at different pH values. All data are presented as the mean ± SD (n=3).

The core-shell nanoparticle GPDC-MSNs (Figure 1A) were prepared by hydrating a biofunctional lipid film containing the lipid material DC, the HCC targeting material Gal-P123 and phospholipids with an MSN colloidal solution. The pH-sensitive charge-switchable lipid DC and Gal-P123 were synthesized as described in our previous report 19, 28. The MSNs were synthesized by a base-catalyzed sol-gel procedure using trimethylbenzene as a pore-enlarging agent. TEM images showed that the synthesized MSNs possess a large pore size, high dispersity and uniform spherical morphology (Figure 1B). The SAXRD pattern (Figure 1C) and N_2_ adsorption-desorption isotherm revealed that the MSNs had a 2D hexagonal mesostructure with large pore sizes (8 nm in diameter) and a large surface area (721 m^2^/g), which allowed a high drug loading efficiency of CPT-11 in the nanoparticles (Figure 1D). For GPDC-MSNs, the lipid material DC, Gal-P123 and phospholipids dissolved in chloroform solution formed monolayers, which self-assembled into solid surface-supported layers during the hydration process. TEM images of GPDC-MSNs directly provided visual evidence of the surface-bound lipid layer. As the DC input in the lipid membrane increased, the size of the resulting GPDC-MSNs decreased, and the zeta potential increased accordingly (Figure 1E), reaching a plateau at a content of 50%. At this composition, GPDC-MSN formation slightly increased the particle size of MSNs from approximately 70 nm to 90 nm, and the zeta potential changed from -28.2 ± 5.6 mV to -12.2 ± 3.5 mV (Figure 1F). When incubated in serum-containing PBS, GPDC-MSNs maintained a more stable size than MSNs over time (Figure 1G). These results demonstrated that lipid membrane coating on the MSNs occurs, which stabilizes MSNs against coalescence in biologically relevant media. The effect of Gal-P123 content in the lipid membrane on the tumor cell targeting ability was also investigated. The cellular internalization of GPDC-MSNs in Huh-7 cells was enhanced as the Gal-P123 content increased and stabilized at 10%, confirming the successful fabrication of GPDC-MSNs with optimal drug loading efficiency, stability and HCC cell targeting ability.

It has been reported that the citraconic amide in DC was stable at both neutral and basic pH values but unstable at acidic pH 30, 31. Thus, the DC in GPDC-MSNs could degrade into DOA under acidic conditions (Figure 1A). Nanoparticle charge conversion was monitored by measuring the zeta potential after incubation at different pH values. Figure 1J shows that the surface charge of GPDC-MSNs increased from -12.8 mV at pH 7.4 to 9.4 mV at pH 5.0, reaching 0 mV at approximately pH 6.5, indicating that DC would be hydrolyzed in the presence of enough H^+^. In contrast, GP-MSNs without the DC coating exhibited constant zeta potential at all pH values investigated. The zeta potential variation of GPDC-MSNs can be attributed to the degradation of DC and the uncovering of amine groups in the acidic environment. As shown in Figure 1K, the exposed primary amine content of GPDC-MSNs increased significantly from 3% at pH 7.4 to approximately 56% at pH 5.0, while no sign of free primary amine was observed in GP-MSNs. The hydrolysis of amide bonds corresponds to the increased positive charge of GPDC-MSNs and might facilitate cell internalization.

### Facilitated cellular uptake and MCS penetration of GPDC-MSNs

**Figure 2:**
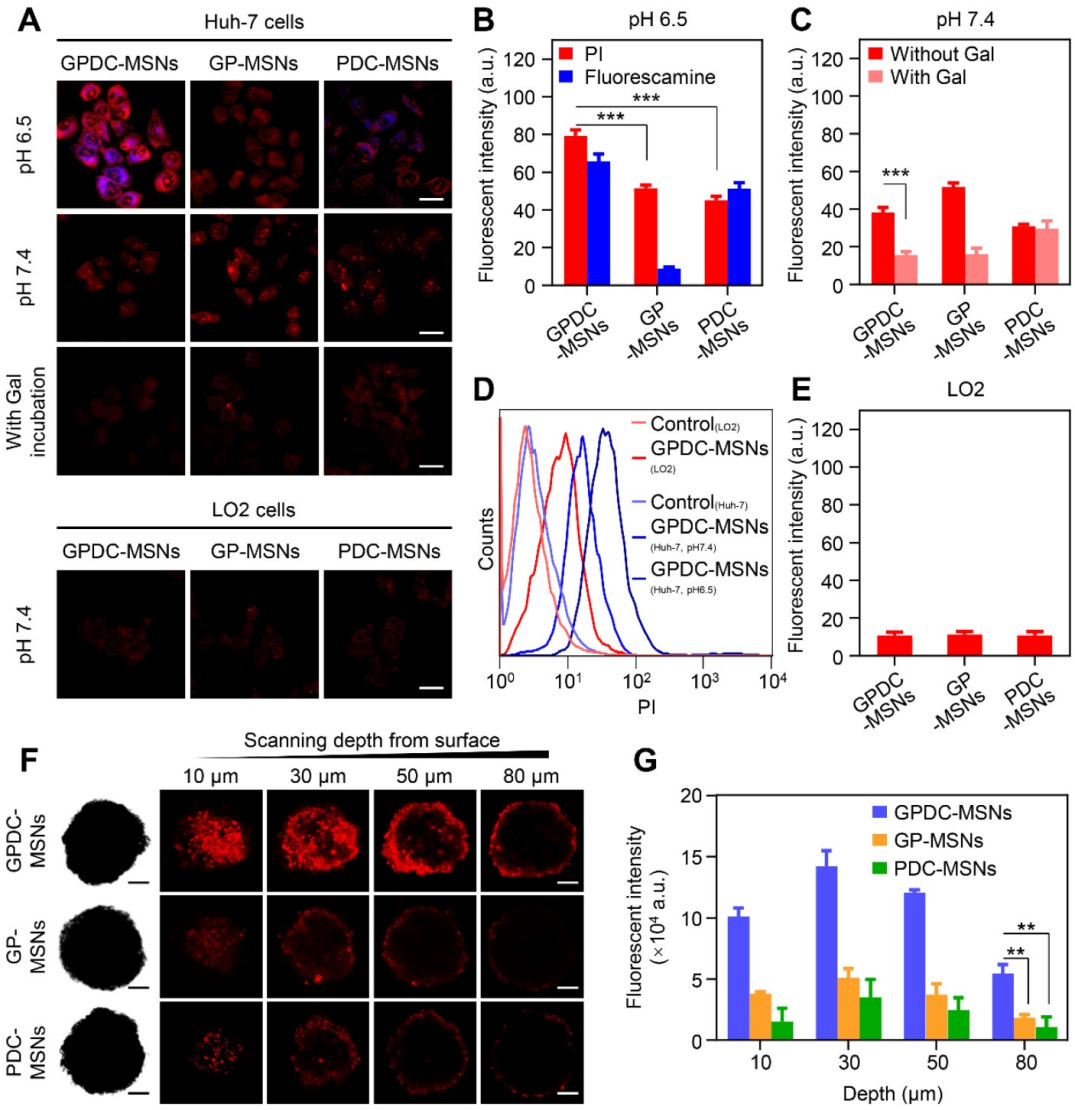
(A) CLSM images of Huh-7 and LO2 cells incubated with GPDC-MSNs, PDC-MSNs and GP-MSNs with or without galactose ligand inhibition. Nanoparticles were labeled with PI (red), and fluorescamine (blue) was added to the culture medium to visualize the amine exposure. Scale bar: 40 μm. (B) Quantitative measurement of the fluorescence intensity in Huh-7 cells by flow cytometry at pH 6.5 and (C) pH 7.4 with or without galactose ligand inhibition. ***p<0.001. (D) Representative flow cytometry histogram of Huh-7 and LO2 cells treated with GPDC-MSNs. (E) Quantitative measurement of the fluorescence intensity in LO2 cells after incubation with DC-MSNs, PDC-MSNs and GP-MSNs. (F) Nanoparticle penetration into the Huh-7 MCSs. Z-stack images were obtained starting from the top and proceeding into the core of the spheroid at intervals of 20 μm. Scale bar: 100 μm. (G) Quantification of the fluorescence intensity in the inside area of MCSs. **p<0.01. All data are presented as the mean ± SD (n=3).

Due to the significant role of surface charge and ligand modification of nanoparticles in cell internalization, the charge conversion property and galactosylation were expected to endow GPDC-MSNs with comparative selectivity in cellular uptake between normal (pH 7.4) and tumor (pH 6.5) environments. Herein, Huh-7 cells with ASGP receptor overexpression were incubated with PI-labeled GPDC-MSNs, PDC-MSNs or GP-MSNs in simulated normal physiological and acidic tumor extracellular environments, respectively. The membrane-impermeable dye fluorescamine was used to visualize the exposed amines of DC and produce blue fluorescence after nanoparticle cellular uptake. As shown in Figure 2A and B, the cellular uptake of GPDC-MSNs at pH 6.5 is significantly higher than that at pH 7.4, and intense fluorescamine fluorescence induced by amine exposure was observed after incubation, indicating acid-triggered enhancement of cellular uptake. In contrast, the internalization of GP-MSNs was not significantly affected by pH, and no blue fluorescence signal was detected. Moreover, the internalization of GPDC-MSNs was approximately 1.8 times that of PDC-MSNs at pH 6.5 due to the interaction of galactosyl with the ASGP receptor on the Huh-7 cell membrane. The marked difference between the cellular uptake of nanoparticles with or without galactose ligand inhibition also suggested receptor-mediated endocytosis (Figure 2C). The GPDC-MSNs at pH 6.5 showed the highest cellular uptake in Huh-7 cells among all groups, which was 7.2-fold higher than that in the normal liver cell line LO2 (Figure 2D and E), indicating superior tumor cell selectivity resulting from the combined effect of acid-triggered and receptor-mediated cellular uptake.

To further evaluate the nanoparticle penetration, MCSs derived from Huh-7 cells were incubated with PI-labeled GPDC-MSNs, GP-MSNs or PDC-MSNs at pH 6.5 (Figure 2F and G). For GP-MSNs and PDC-MSNs, red fluorescence was mostly detected on the periphery of MCSs at a scanning depth of 30 μm. In contrast, the penetration capability of the GPDC-MSNs was significantly greater, and red fluorescence inside the MCSs could be clearly observed even at a scanning depth of 50-80 μm. Quantitative analysis indicated that more than 3-fold higher red fluorescence intensity was detected in GPDC-MSNs treated MCSs than in GP-MSNs or PDC-MSNs treated MCSs. It has been reported that the intratumoral acidic environment is not spatially uniform, but a pH gradient exists, decreasing from 7.4 to 6.0 32. The core of a solid tumor has a lower pH due to the hypoxia-triggered acceleration of glycolysis 33. The charge-converting lipid DCs could gradually degrade under the pH gradient as nanoparticles migrate to the tumor core to expose positively charged amino groups. GPDC-MSNs showed a superior tumor penetration effect, which could be attributed to the enhanced cellular uptake mediated by gradual charge activation and galactosyl targeting and may enhance the antitumor effect in deep tumor areas.

### Triggered release of therapeutics in endosomal pH

**Figure 3:**
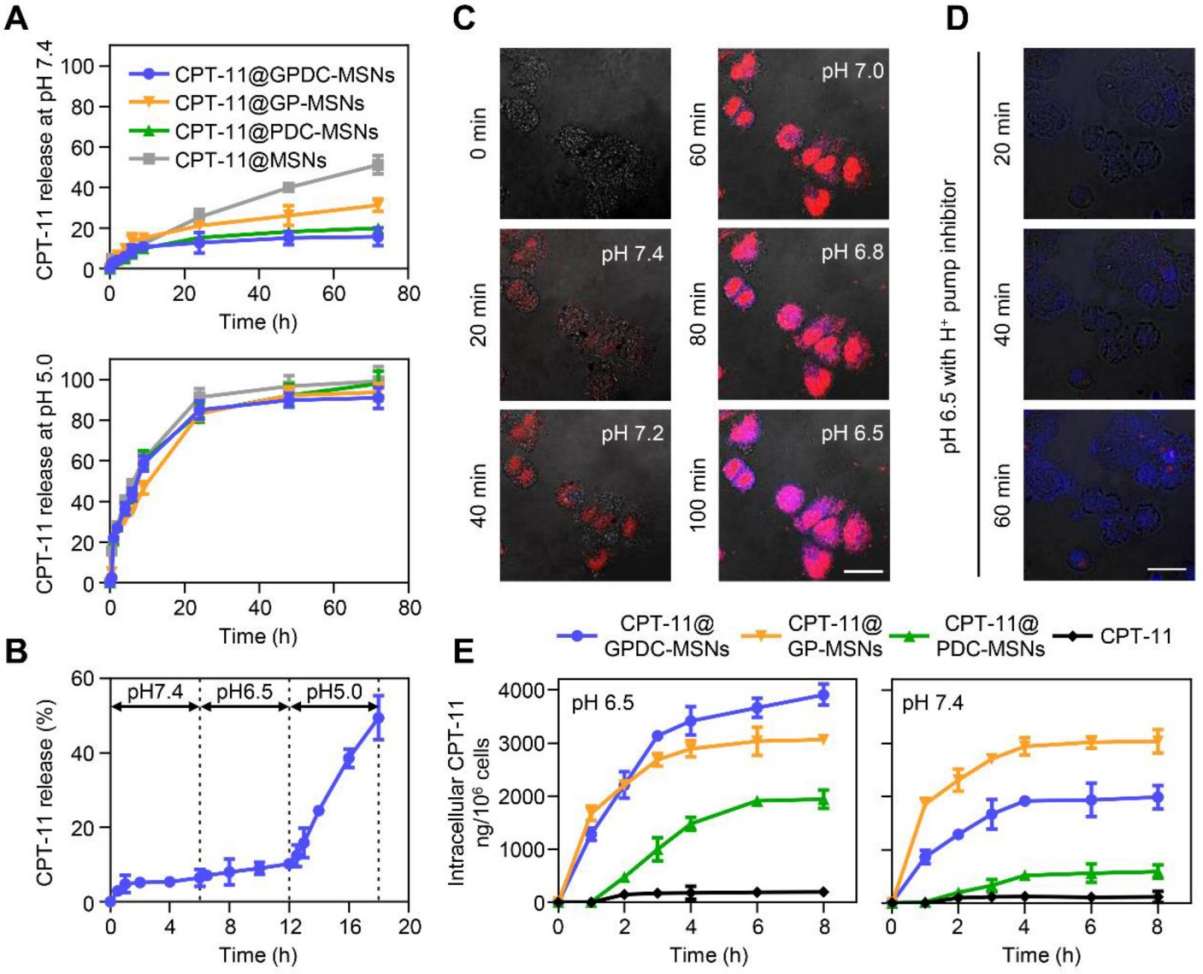
(A) CPT-11 release from CPT-11-loaded MSNs, PDC-MSNs, GP-MSNs and GPDC-MSNs when exposed to simulated body fluid (pH 7.4) and acidic endosome/lysosome buffer (pH 5.0) at 37 °C. (B) Drug release from CPT-11@ GPDC-MSNs upon lowering the pH to 5.0 after exposure to pH 7.4 and pH 6.8 buffers for 12 h. (C) Real-time confocal microscopy images monitoring intracellular cargo release in Huh-7 cells after incubation with PI-labeled GPDC-MSNs at a pH gradient from 7.4 to 6.5. After recording at pH 7.4 for 20 min, the pH of the incubation medium was adjusted to 7.2, 7.0, 6.8 and 6.5 at 20 min intervals. Fluorescamine was added to the culture medium to visualize the amine exposure. Scale bar: 40 μm. (D) Real-time images of the intracellular cargo release of PI-labeled GPDC-MSNs in Huh-7 cells after bafilomycin A1 (an endosome/lysosome acidification inhibitor) treatment. Scale bar: 40 μm. (E) Quantification of CPT-11 accumulation in Huh-7 cells after various treatments at pH 6.5 and pH 7.4. All data are presented as the mean ± SD (n=3).

Ideal nanoparticles should provide intracellular release of encapsulated drugs to enhance therapeutic efficacy and should prevent premature drug leakage in physiological environments to avoid toxicity to healthy tissues. The *in vitro* release of CPT-11 from nanoparticles (Figure 3A) showed that GPDC-MSNs could minimize premature drug leakage in simulated body fluid (pH 7.4), whereas MSNs with no supported lipid layer leaked more than 50% of the encapsulated CPT-11 within 72 h. This premature release of cargo by MSNs might result in off-target toxicity to normal bystander cells. Exposing GPDC-MSNs to a pH 5.0 buffer (simulated endosomal pH), 85% of encapsulated CPT-11 was released within 24 h. This may be attributed to the destabilization of the supported lipid layer in the acidic environment, promoting rapid release of the loaded drugs. In addition, the pH of the solution was lowered to pH 5.0 to confirm that CPT-11 release from GPDC-MSNs was indeed triggered by pH. The release of CPT-11 in simulated body fluid (pH 7.4) and tumor microenvironment (pH 6.5) conditions was monitored for 12 h, followed by a lowering of the pH of the dissolution medium to 5.0. As shown in Figure 3B, the release profiles in pH 6.5 buffer followed a similar trend to that in pH 7.4 buffer, with little release of the encapsulated drug. When the pH changed to 5.0, a burst release of CPT-11 was observed. The responsive drug release of GPDC-MSNs potentially matched biological systems and avoided off-target toxicity.

To further monitor the intracellular cargo release process, Huh-7 cells were incubated with PI-labeled GPDC-MSNs, and fluorescamine was added to visualize the amine exposure. PI was used as a fluorescence dye to visualize the intracellular cargo release due to its emitting a fluorescent signal only when released from nanoparticles in the cells. It has been reported that although the average extracellular pH within tumors is generally acidic, the pH environment is not spatially uniform. Intratumoral pH mapping is affected by the distance from tumor blood vessels, and pH gradients exist within tumors, decreasing from 7.4 to approximately 6.5 34, 35. In this experiment, the pH of the culture medium was gradually adjusted from 7.4 to 6.5 to stimulate the tumor extracellular pH gradient, and live cell images were collected for 100 min. Figure 3C shows that the cells emitted a low fluorescent signal at pH 7.4 after treatment with GPDC-MSNs for 20 min. However, notable drug release was observed as the pH gradually decreased to 7.2, 7.0, 6.8 and 6.5. The blue fluorescence produced by fluorescamine combined with GPDC-MSNs confirmed the exposed amine and internalization of nanoparticles in cancer cells when cultured at acidic pH.

To further investigate the role of endosomes/ lysosomes in intracellular drug release, the acidification of endosomes/lysosomes was inhibited using bafilomycin A1 (Figure 3D). Huh-7 cells were treated with PI-labeled GPDC-MSNs at pH 6.5, with fluorescamine added to the culture medium. After bafilomycin treatment, cells gradually produced blue fluorescence (fluorescamine conjugated amine) over time, indicating the surface charge conversion and internalization of nanoparticles. However, no red fluorescence (PI) was detected during incubation, which indicated that bafilomycin may have prevented endosome/lysosome acidification and completely inhibited drug release from the mesoporous structure. For GPDC-MSNs without bafilomycin treatment, both PI and fluorescamine fluorescence were observed, indicating rapid internalization and intracellular fluorescent release. Thus, the intracellular release factor was the acidic endosome/lysosome system in tumor cells.

To demonstrate that GPDC-MSNs facilitated drug accumulation within tumor cells, the intracellular concentration of CPT-11 in Huh-7 cells at pH 6.5 and 7.4 was studied after the application of various CPT-11 formulations. Figure 3E shows that CPT-11@GPDC-MSNs produced a higher intracellular CPT-11 concentration than free CPT-11 (19.6-fold), CPT-11@PDC-MSNs (2.0-fold) and CPT-11@GP-MSNs (1.3-fold) in Huh-7 cells at pH 6.5 after 8 h of incubation. This might be explained by galactosyl targeting and charge conversion-induced cell internalization as well as the intracellular burst drug release of GPDC-MSNs. Of note, GPDC-MSNs showed a lower intracellular drug concentration than GP-MSNs (p<0.05) at pH 7.4, which may be due to the DC forming a negatively charged barrier on the surface of GPDC-MSNs at normal tissue pH, thereby preventing cell internalization. Thus, GPDC-MSNs could significantly promote intracellular triggered drug storm release and avoid premature leakage in normal tissues, leading to increased antitumor efficacy and reduced systemic toxicity.

### Cytotoxicity and cell apoptosis

**Figure 4:**
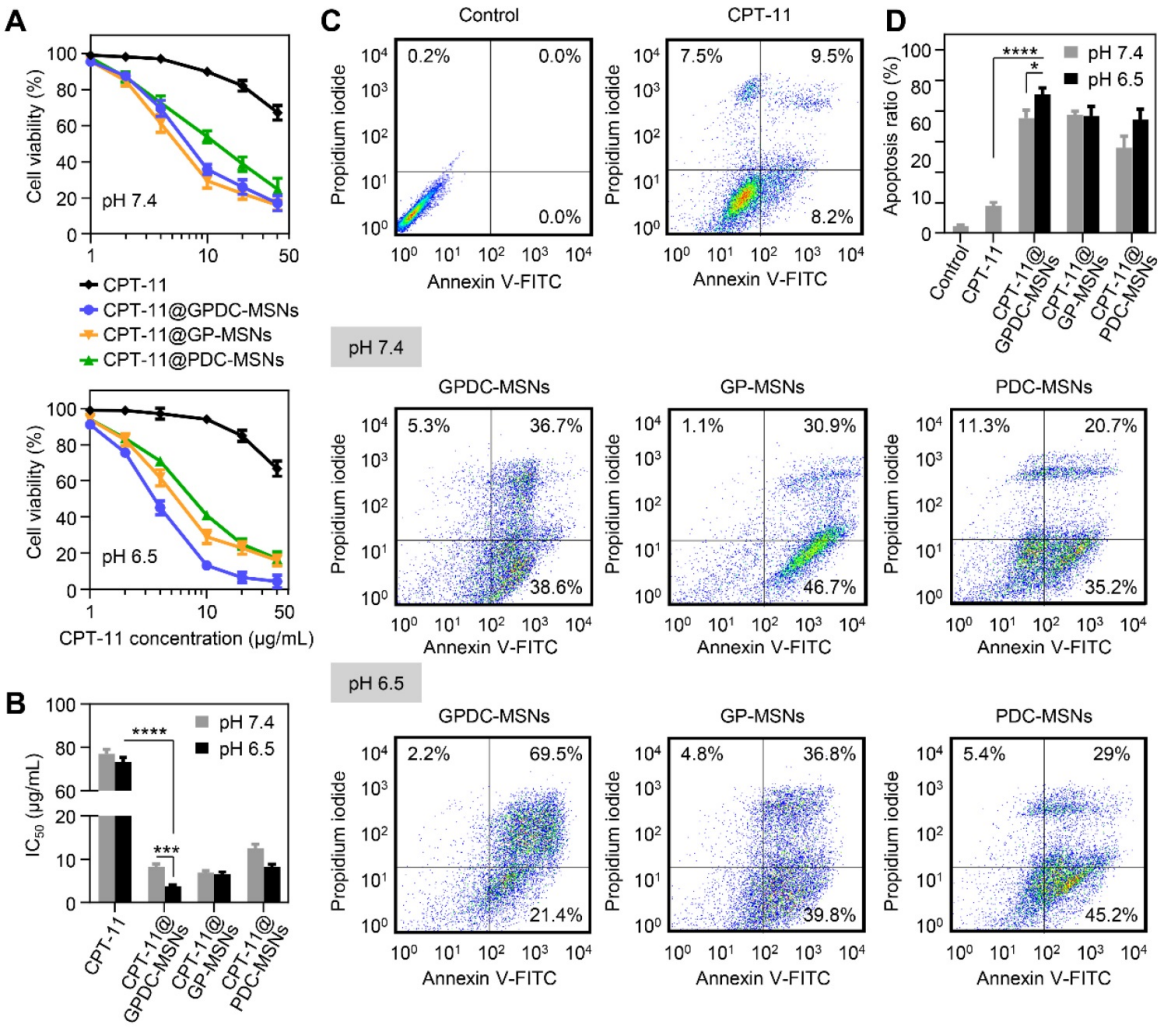
(A) Cell viability of Huh-7 cells after incubation with CPT-11 formulations at pH 7.4 and pH 6.5. (B) IC_50_ values of CPT-11 formulations in Huh-7 cells after exposure to pH 7.4 and pH 6.5 culture media. ***p<0.001, **** p<0.0001. (C) Flow cytometric examination and (D) quantitative analysis of Huh-7 cell apoptosis after different treatments for 24 h. Early apoptotic cells appeared in the lower right quadrant, and late apoptotic cells appeared in the upper right quadrant. *p<0.05, **** p<0.0001. All data are presented as the mean ± SD (n=3).

The cytotoxicity of CPT-11-loaded GPDC-MSNs was investigated in Huh-7 cells by MTT assay, and the IC_50_ value was calculated simultaneously. As shown in Figure 4A and B, the CPT-11 formulations exhibited dose-dependent antiproliferative effects on Huh-7 cells. CPT-11-loaded nanoparticles demonstrated better antitumor activity than free CPT-11 (IC_50_ 70 μg/mL). Treatment with GPDC-MSNs significantly increased the cytotoxicity of CPT-11 by 19.5-fold at pH 6.5 due to the high cellular internalization and intracellular rapid release of the encapsulated drug. Moreover, CPT-11@GPDC-MSNs showed pH-dependent toxicity, and the cell viability was 5.2% after treatment with 40 μg/mL for 72 h at pH 6.5 (IC_50_ value 3.8 μg/mL), which was 2.2-fold lower than the IC_50_ at pH 7.4. This was attributed to the charge conversion of GPDC-MSNs at simulated acidic tumor extracellular matrix pH facilitating cellular internalization. No significant differences were observed when Huh-7 cells were treated with GP-MSNs at pH 6.5 and 7.4.

Huh-7 cells were incubated with free CPT-11, CPT-11-loaded GPDC-MSNs, PDC-MSNs and GP-MSNs at a drug concentration of 40 μg/mL for 24 h and then stained with Annexin V and PI to measure cell apoptosis. Figure 4C and D shows that free CPT-11 did not induce marked cell apoptosis (17.7% apoptosis at 24 h), which was consistent with the cytotoxicity results. The delivery of CPT-11 into Huh-7 cells by GPDC-MSNs increased cell apoptosis to 90.9% at pH 6.5, which was higher than that resulting from treatment at pH 7.4 (75.3%). CPT-11@PDC-MSNs and CPT-11@GP-MSNs significantly increased cell apoptosis, suggesting that both the targeting ligand and charge conversion lipid in the nanoparticles were essential for promoting cell apoptosis. In addition, it has been reported that CPT-11 is a typical substrate of breast cancer resistance protein (BCRP), which showed high expression in Huh-7 cells and played an important role in drug resistance. Gal-P123 has been demonstrated to show an inhibitory effect on BCRP-mediated drug efflux in our previous study 36. Therefore, Gal-P123 in GPDC-MSNs could further increase the intracellular accumulation of CPT-11 in Huh-7 cells and enhance the cytotoxicity and apoptosis of tumor cells.

### *In vivo* pharmacokinetics and tumor targeting

**Figure 5:**
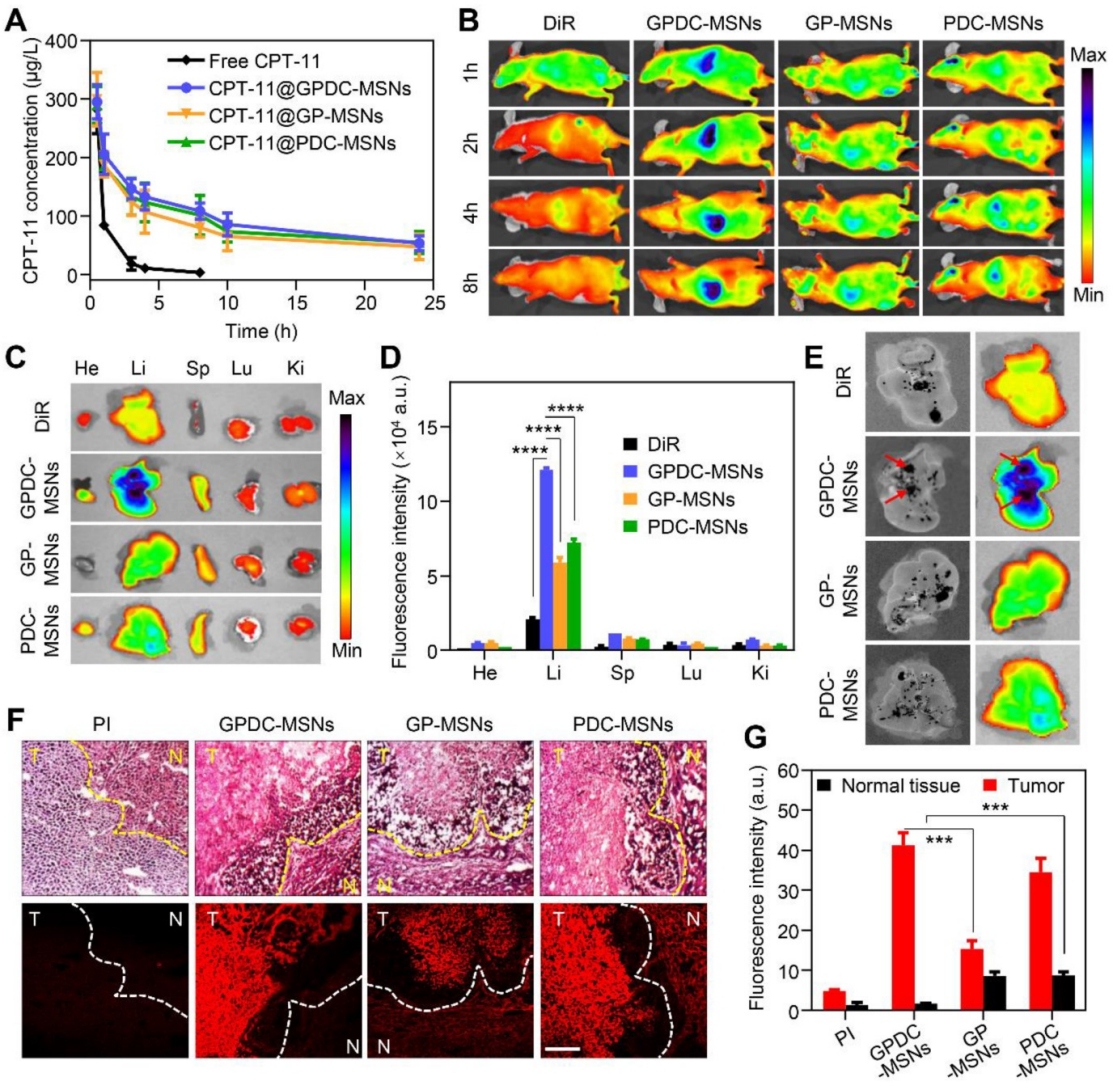
(A)* In vivo* pharmacokinetics of free CPT-11, CPT-11 loaded GPDC-MSNs, PDC-MSNs and GP-MSNs. (B) Fluorescence images of mice treated with DiR solution, DiR-labeled PDC-MSNs, GP-MSNs and GPDC-MSNs. Images were taken 1, 2, 4, and 8 h after intravenous administration. (C) *Ex vivo* fluorescence images and (D) quantitative biodistribution of important organs excised 8 h post injection. ****p*<*0.0001. (E) Images of the excised HCC-bearing liver showing the colocalization of tumor tissues and nanoparticles. The black patch in the liver is HCC tissue (left in every image), and the fluorescence images show the distribution of DiR-labeled formulations (right in every image). (F) Liver sections and (G) quantitative analysis of fluorescence intensity showing PDC-MSNs, GP-MSNs and GPDC-MSNs accumulation in the interior of HCC and normal hepatic cells. N: normal hepatic tissue; T: tumor tissue. Original magnification: 200×, scale bar: 50 μm. ***p*<*0.001. All data are presented as the mean ± SD (n=3).

To evaluate the* in vivo* circulation behavior of GPDC-MSNs, SD rats were used as the animal model and i.v. injected with free CPT-11, CPT-11-loaded GPDC-MSNs, PDC-MSNs and GP-MSNs. As shown in Figure 5A, CPT-11-loaded GPDC-MSNs, PDC-MSNs and GP-MSNs showed longer circulation times than the free CPT-11 group, probably due to the immune-evasion ability of the hydrophilic polymer Pluronic P123. The elimination half-life (t_1/2_) of CPT-11@GPDC-MSNs was ~13 h compared to the rapid clearance of free CPT-11 with a short t_1/2_ of ~1.5 h. The area under the curve (AUC) of CPT-11@GPDC-MSNs was found to be approximately 5.5-fold higher than that of free CPT-11. The results indicated that Gal-P123 plays an important role in the stealth capability of in GPDC-MSNs *in vivo*. Thus, the prolonged systemic circulation might synergize with the homing ability of galactosyl to improve the tumor delivery of the therapeutic agent.

GPDC-MSNs were expected to enhance the HCC targeting effect *in vivo* due to the active targeting ligand Gal-P123 and the charge conversion property of DC. The *in vivo* tumor-targeting efficacy was tracked using noninvasive NIRF imaging. Figure 5B shows that after the intravenous administration of near-infrared dye DiR-labeled formulations, a strong NIRF signal was observed in the whole body, and this signal gradually distributed over time. The DiR solution had a nonspecific distribution (as measured by fluorescence) within the body, and the fluorescence decreased rapidly post injection. In contrast, DiR-labeled GPDC-MSNs progressively accumulated in the liver more than did f the PDC-MSNs and GP-MSNs. In addition, the fluorescence signal in the removed internal organs (heart, liver, spleen, lung and kidney) 8 h post injection (Figure 5C, D) indicated that GPDC-MSNs significantly enhanced the accumulation in the liver compared to DiR solution (5.8 times), PDC-MSNs (1.7 times) and GP-MSNs (2.1 times). By comparing the location of orthotopic hepatic tumors to nanoparticle accumulation, strong fluorescence was observed to colocalize with the tumor tissue after GPDC-MSNs treatment (Figure 5E), indicating that GPDC-MSNs could selectively target HCC tissue.

We further studied the tumor targeting and cellular location of nanoparticles in Huh-7 tumors with histological analysis in excised livers. PI was used to label the mesoporous core of nanoparticles to confirm the location of integrated nanoparticles along with the corresponding biodistribution results. Four hours after the intravenous injection of PI-labeled nanoparticles, livers with HCC tissue were detached and sectioned for H&E staining. As shown in Figure 5F, tumor cells, tumor boundaries and normal cells could be distinguished in the H&E-stained liver sections. The fluorescence signal of GPDC-MSNs in interior tumor tissues was up to 25 times higher than that in normal hepatic tissue (Figure 5G), suggesting that the charge conversional nanoparticles have superior selectivity towards HCC cells. Moreover, GPDC-MSNs showed the highest distribution in HCC tissue but the lowest concentration in normal tissue among the groups, and these observations agreed well with the results of the *in vitro* cell uptake experiment. These results demonstrated that GPDC-MSNs exhibited HCC targeting and selective internalization in tumor cells, which could be attributed to pH-triggered charge conversion and ASGP-mediated endocytosis.

### Enhanced antitumor efficacy of CPT-11@GPDC-MSNs

**Figure 6:**
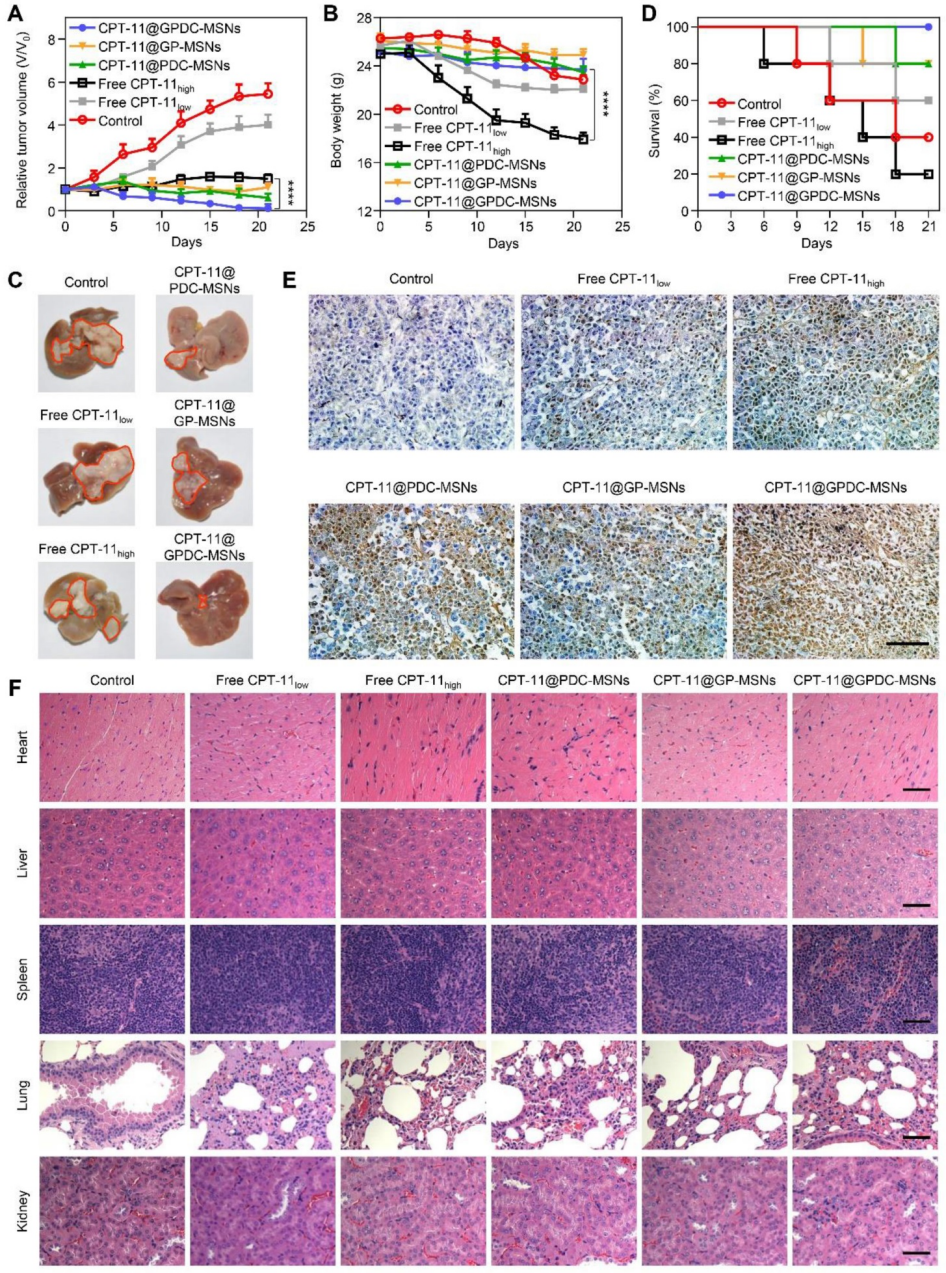
*In vivo* antitumor efficacy of CPT-11 formulations in Huh-7 ectopic and orthotopic xenograft tumor models. (A) Tumor growth curve and (B) average body weight of mice bearing ectopic Huh-7 tumors treated with free CPT-11, CPT-11-loaded PDC-MSNs, GP-MSNs or GPDC-MSNs. ****p*<*0.0001. (C) Representative liver photographs, (D) overall survival, (E) TUNEL assay of HCC tissue and (F) H&E staining of major organs in mice bearing Huh-7 orthotopic tumors after treatment with CPT-11 formulations. scale bar: 50 μm. All data are presented as the mean ± SD (n=6).

The advantages of GPDC-MSNs in CPT-11 delivery may enhance cancer therapy *in vivo*. Therapeutic efficacy was evaluated using both ectopic and orthotopic Huh-7 HCC models after the intravenous injection of different CPT-11 formulations. Figure 6A shows that compared to the control, tumor growth was inhibited in all groups treated with CPT-11 formulations in a dose-dependent manner. CPT-11@GPDC-MSNs decreased tumor volumes the most. Since cancer therapy must also include considerations of patient quality of life (such as weight loss), we measured the body weights of HCC-bearing mice (Figure 6B). The control group (saline-treated only) lost weight quickly and died early. Although free CPT-11 (100 mg/kg) significantly inhibited tumors compared to the control, it also reduced body weight. We attributed this to the severe diarrhea observed in the high-dose free CPT-11 treatment group 37. Interestingly, even at a dose of 10 mg/kg, CPT-11@GPDC-MSNs showed more efficient tumor inhibition than higher-dose free CPT- 11, and these lower doses had less systemic toxicity, reflected in smaller changes in body weight. CPT-11@ GPDC-MSNs also demonstrated superior suppression of orthotopic Huh-7 tumor nodule growth on the surface of the liver (Figure 6C), and the median survival was significantly extended compared to 13-15 days for the control and high-dose free CPT-11 treatment groups (Figure 6D). Notably, no mice died during the 21-day treatment with CPT-11@GPDC- MSNs. The enhanced HCC tissue targeting, selective accumulation in tumor cells and stimulated intracellular drug release of GPDC-MSNs contributed to their improved antitumor efficacy *in vivo*.

Furthermore, H&E and TUNEL staining were performed after treatment. As shown in Figure 6E, tumor tissue treated with normal saline was malignant. Cancer cell nuclei were large and oval-shaped with more chromatin and visible binucleates. In contrast, tumor sections treated with CPT-11@GPDC-MSNs underwent apoptosis and significant tissue loss across a large tumor area. Notably, cell apoptosis of CPT-11@GP-MSNs was deficient in oncotherapy, probably due to insufficient internalization into tumors. The H&E staining assay of the major organs, including the liver, spleen, heart, lungs and kidneys, appeared normal (Figure 6F), indicating the minimal systemic toxicity and high safety of CPT-11@GPDC-MSNs.

## Conclusions

A key goal in cancer therapy is to achieve specific accumulation at the tumor site and on-demand drug release in the tumor cells, thus enhancing the therapeutic efficacy and decreasing side effects. In this study, we designed tumor-targeted nanoparticles, GPDC-MSNs, that underwent stepwise responses in the tumor environment. The acid-sensitive lipid DC and Gal-P123 in GPDC-MSNs contribute to selective HCC targeting and cellular internalization through pH-responsive charge conversion and ASGP receptor-mediated endocytosis. In addition, GPDC-MSNs retained the drug in the blood circulation (pH 7.4) but triggered release of the encapsulated CPT-11 in the tumor cells. Both *in vitro* and *in vivo* studies demonstrated that stepwise- responsive GPDC-MSNs exerted outstanding antitumor efficacy against HCC and simultaneously exhibited less systemic toxicity than free CPT-11. Intelligent nanoparticles were prepared using biocompatible materials via a simple single-step assembly method, which may have advantages in synthesis for clinical trials. We also believe that this stepwise-responsive strategy will provide opportunities to explore smart drug delivery nanoplatforms for HCC targeting and therapy in the future.
